# *“I am alive because of her”:* factors affecting adherence to combination antiretroviral therapy among people living with HIV in KwaZulu-Natal, South Africa

**DOI:** 10.1186/s12879-022-07667-x

**Published:** 2022-08-08

**Authors:** Marian Loveday, Jennifer Furin, Sindisiwe Hlangu, Tasneem Naidoo

**Affiliations:** 1 HIV Prevention Research Unit, South African Medical Research Council, Durban, KwaZulu-Natal South Africa; 2 Department of Global Health and Social Medicine, Harvard Medical School, 641 Huntington Ave., Boston, MA 02115 USA; 3 R. K. Khan Hospital HAST Unit, Department of Health, Durban, KwaZulu-Natal South Africa; 4 CAPRISA-MRC HIV-TB Pathogenesis and Treatment Research Unit, Doris Duke Medical Research Institute, University of KwaZulu-Natal, Durban, South Africa

**Keywords:** South Africa, HIV, Antiretroviral therapy, Adherence

## Abstract

**Background:**

People living with HIV need to take lifelong, combination antiretroviral therapy (cART), but there have been only limited explorations of how factors affecting adherence can change over the course of an individual’s lifetime.

**Methods:**

We carried out a qualitative study of men and women living with HIV in KwaZulu, Natal, South Africa who were prescribed cART and who had periods of higher and lower adherence.

**Results:**

18 individuals participated in open-ended interviews. Using a dynamic theory of adherence, we identified factual, relational, and experiential factors that were associated with adherence and non-adherence to cART. Periods of non-adherence were commonly reported. Participants described relationships and experiences as being important influences on their ability to adhere to cART throughout their treatment journeys.

**Conclusions:**

Periods of non-adherence to cART are common. While many cART counseling models are based on conveying facts to people prescribed cART, providing opportunities for supportive relationship where people can process their varied experiences is likely important to maintaining health for people living with HIV.

**Supplementary Information:**

The online version contains supplementary material available at 10.1186/s12879-022-07667-x.

## Background

In 2020, there were an estimated 37.7 million people living with HIV [1], all of whom could potentially experience healthy lifespans if they are started and maintained on effective combination antiretroviral treatment (cART). This will require testing and initiating on treatment an additional 12 million people as well as supporting the 25.7 million people on cART to be able to continue taking their therapy. In order to be effective, cART must be taken as part of a lifelong commitment through many different events, phases, and experiences.

A significant body of literature exists on factors affecting adherence to HIV, with a heavy emphasis on individual-level factors that may contribute to challenges in taking cART as it is prescribed [2]. While such understandings have been important in identifying and overcoming some challenges to adherence—including pill burden, adverse events, and lack of factual information about both HIV and cART—they often do not take into account social, contextual, and economic barriers to adherence [3]. A growing body of literature suggests that these larger forces—often located outside the individual—play a significant role in cART adherence over the course of a life [4]. Even these more comprehensive explorations of cART, however, tend to view adherence as a more “static” concept rather than a fluid state into which people living with HIV may move in or out of depending on situations and circumstances they are experiencing [5].

Because cART must be taken over the course of a lifetime, understanding the treatment journey taken by someone living with HIV—including factors that promote or detract from adherence—is essential so that ideal support can be provided. For this reason, we undertook a qualitative study with persons who were on cART in which they were asked to share their illness and treatment narratives. The study focused on people living with HIV in South Africa who had been prescribed cART but who also had a detectable serum HIV viral load at some point in the past 12 months, since it is likely such individuals were experiencing challenges with cART adherence during this time. This population was selected to allow for a comprehensive evaluation of periods of adherence and non-adherence within the same individual, and we report the results of this qualitative study here.

## Methods

### Study design

This was a qualitative study done using open-ended interviews. Individuals older than 18 years, who had failed to collect their medication in the last 6 months, in addition to having a detectable viral load at some point in the past 12 months were identified in the ART clinic register at a regional hospital in KwaZulu-Natal. Individuals were purposively selected to ensure both males and females were represented.

### Study setting and population

The study was conducted at the RK Khan hospital, in a peri-urban setting in KwaZulu-Natal South Africa over 4 months, from June 1 to September 30, 2021. RK Khan hospital is a regional hospital with 543 beds with a busy ART clinic providing HIV management for the local population as well as serving as a referral centre for a broader area. Between January 1 and December 31, 2021, 136 individuals with HIV were started on cART at the hospital and a further 141 patients were transferred in for management of HIV complications (e.g., renal impairment, drug induced liver injury secondary to cART, second line regimen failure and rifampicin-resistant TB) from their approximately 30 referral clinics.

### Data collection and analysis

An initial 92 individuals were identified as potentially eligible for the study, but only 40 could be contacted. A total of three declined participation in the study. The first, a truck driver, was only available on weekends; the second, a nurse in the hospital was too busy at work and the third, having initially agreed to participate, could not be contacted. In total, 18 people participated in open-ended interviews using a semi-structured guide (See Additional file 1: Appendix 1) in a private office setting with no other individuals present. The interview guide was designed to ask about their experiences with taking cART over their lifetimes. All interviews were conducted by one female author (SH) who has more than a decade of experience in open-ended interviewing) in the language in which the participant felt most confident (isiZulu or English). The interviews were recorded and transcribed into English for analysis. Participants were told about the interviewers’ interest in the topic matter as part of the formal consent process and that the interviewer was employed as a full-time researcher. Each participant was interviewed only once with interviews lasing between 30 and 90 min. Field notes were not kept or analyzed.

### Theoretical approach

We utilized a dynamic theory of cART adherence developed by Eshun-Wilson and colleagues to ground our data analysis [6]. This model was based on a qualitative systematic review of more than 59 studies of adherence to cART done in Africa in which a theoretical model of cART adherence was developed in which factors external to the person on cART (such as poverty, stigma, conflicting facts, social identity, and health care provider behaviors) interacted with internal “motivational” factors (such as levels of self-efficacy, acceptance of HIV status, previous or current HIV-related illness, and family/social responsibilities) to form a “tipping point” driving the person into adherence or non-adherence.. The model helped inform the interview questions. We attempted to build upon this model as well as exploring how this tipping point might change within the same individual over the course of a lifetime. The literature has explored how facts and knowledge about HIV and cART can contribute to adherence but also how experiences with HIV and interactions with others can impact adherence as well [7]. Thus we explored these domains (referring to them as “factual”, “experiential”, and “relational” throughout the results section. To build upon the model developed by Eshun-Wilson and colleagues, we also utilized a social-ecological model [8] which considers complex and changing interactions at a social and ecological level as contributing to periods of adherence and non-adherence over a lifetime.

### Reflexivity

As part of the importance practice of reflexivity, which is essential in qualitative research, our team acknowledges that as some of us are health care providers, we may have had biases regarding the experiences of the individual participants. As an all-female research team, we also recognize that our gender may have had an impact on the interview process, data analysis, and write-up of the results. We discussed these areas with one another throughout the research project.

### Ethical approval and consent to participate

Written informed consent was obtained from all the patients willing to participate in the study. Written informed consent was provided by all study participants. The consent included participation in the interview and digital audio recording, the voluntary terms of involvement in the study and the assurance of confidentiality and anonymity. Patient anonymity was maintained by identifying each patient using a unique identification number. Ethical approval was obtained from the South African Medical Research Council (SAMRC) Ethics Review Committee (ED050-11/2020) and the KwaZulu-Natal Health Research Committee. This research was carried out and approval and informed consent were obtained in accordance with the World Medical Assembly (WMA) Declaration of Helsinki—Ethical Principles for Medical Research Involving Human Subjects [9].

### Role of the funding source

This work was funded by the Bill and Melinda Gates Foundation (INV-004597). The funders were not directly involved in the data collection, analysis, write up or review in any way.

## Results

### Participant characteristics

A total of 18 individuals participated in the interviews, and their demographics are summarized in Table 1. The average age was 42 years, and seven participants were males. Five participants had been living with HIV for longer than 15 years. The three participants diagnosed as HIV-positive between 1994 and 2002 had to wait several years before they were eligible for cART. (Although cART was available in South Africa beginning in late 2004, it was initially only available to those with a very low CD4 count. This threshold increased over the years.)

**Table 1:** Demographic characteristics of study participants

Study ID	Age	Gender	Time on cART
001	62	Female	~ 5 years
002	19	Female	19 years
003	51	Female	5 years
004	47	Male	3 years
005	39	Female	9 years
006	27	Female	4 years
007	42	Female	11 years
008	57	Male	11 years
009	42	Male	4 years
010	54	Female	7 years
011	25	Female	5 years
012	39	Male	2 years
013	57	Male	9 years
014	23	Female	17 years
015	48	Female	17 years
016	36	Male	Months
017	41	Male	15 years
018	40	Female	4 years

### Reasons for delayed treatment initiation

Although the study initial was planned to assess factors associated with adherence to cART, the open-ended interviews revealed that even when eligible to begin cART, some participants did not start on recommended treatment. For example, one participant delayed initiating cART for 11 years and one participant for 8 years. These participants described how it was only when they became ill, often sick enough to become hospitalised, that they initiated treatment. In contrast, one participant who was ill at the time of diagnosis started treatment immediately.

Several reasons were given for delayed treatment initiation. First was the shock of receiving an HIV diagnosis, especially among people who tested as part of a work or community screening program as opposed to those who tested because they were feeling unwell. As one participant who delayed starting cART reported:

*“Yes, the first time they told me that I am HIV positive, I started out not believing it, I thought the doctor must have mistaken. How am I positive?”* (Participant 9).

Another common reason for delayed treatment initiation was feeling physically well at the time of diagnosis. As another participant reported:

*“It had been a while since I had known (that I had HIV) but there was nothing that was giving me an issue and my immune system was alright.”* (Participant 10).

And another reported:

*“When I was working for Telkom, people from [the HIV testing program] came to test us and they told me I was HIV positive, they then arranged for me to see a doctor. I don’t want to lie I was afraid, and I did not take note of it… during that time, I didn’t have any problem. I went to start treatment (later) because I could feel my body getting weaker and everything.”* (Participant 13).

Participants also reported delaying treatment initiation for fear of adverse events, because they wanted more time to make a decision about their HIV treatment options, or because they started on treatment for tuberculosis (TB) first and thus their cART initiation was delayed.

### Overarching themes for non-adherence once cART initiated

**Fig. 1:**
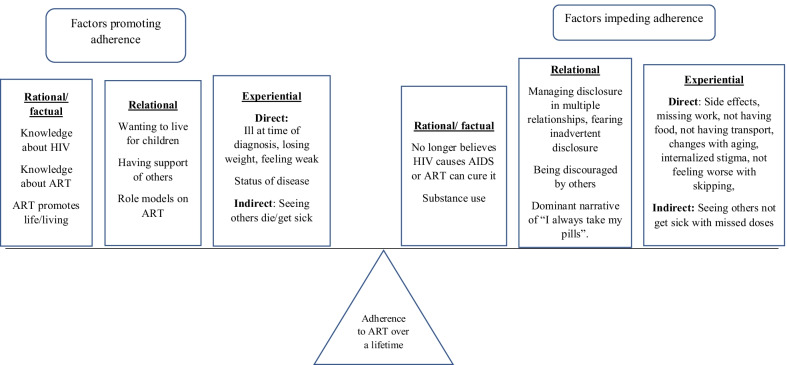
Adult HIV Treatment Journey: Analytic Framework

A number of thematic areas emerged as part of the qualitative analysis, and participants tended to report them as either contributing to their ability to adhere to cART or making it challenging to adhere to cART. Overall, these tended to fall into the larger themes of the “factual,” the “relational,” and the “experiential” realms. Each of these will be described in more detail below, including important sub-themes, and the ways they impact adherence to cART (see Fig. 1).

#### Factual influences

Participants described certain “facts”, knowledge, and relatively objective information that influenced whether or not they took their cART medications as prescribed. We have categorized these as “factual” influences on adherence. Factual influences supporting adherence included: (1) understanding that HIV can be diagnosed via a blood test; (2) that HIV was responsible for the clinical symptoms the person was experiencing; (3) that cART is the best way to keep HIV “under control”; and (4) that cART needs to be taken in a specific way over the course of a lifetime. Factual influences or “misunderstansings” making it difficult to adhere to cART that stood in contrast to these included: (1) that a blood test cannot be used to make a diagnosis of HIV; (2) that HIV was not the cause of the symptoms the participant was experiencing; (3) that cART is not a treatment for HIV; and/or that cART does not need to be taken. Substance use—in which persons described their inability to think “rationally” and comprehend facts because they were impaired—was another important influence on adherence to cART as were mental health challenges.

Most participants recalled that the counseling they received was primarily based on conveying facts:

As one participant noted:

“*They explained that if you have this virus it doesn’t mean that you are going to die soon, and it’s all dependent on how well you take care of yourself. They explained that if you don’t take good care of yourself that’s when the problem starts. They said that if you take your treatment, collect it on time and take it according to how you have been instructed you can live for a long time.”*

The importance of these factual aspects of cART as part of ongoing adherence was discussed by a participant who did not believe he had HIV or needed to take cART to stay healthy. In talking about his HIV diagnosis and adherence challenges, he reported:

*“You see… not that I am denying that I have HIV. I may have it, it’s just that I have never tested for it like when I’m sober or like when I am myself you see…I don’t believe in the ARVs, personally, I don’t believe in them. So, there were times when I would collect the pills but not take them for about three months. But then I would be thinking that maybe they are right about the pills you see. I was on-and-off with them, because I had not decided what to believe about these pills.”*

Some participants reported that their challenges with adherence were often due to their inability to remember to take the medications, noting that this was worse for them early in the course of their treatment. As one participant stated:

*“Sometimes I would forget to take them because it was something unusual to me. You forget, I would forget mostly on weekends, maybe if I had gone out with my friends. I’d forget and remember the next morning, that I didn’t take my pill the previous day.”*

The issue of alcohol and other drug/substance use was reported by several participants as being something that made adherence challenging. Participants often stated that the use of alcohol and/or other substances took away their ability to think rationally or to apply “the facts” they had been given during their treatment journeys. As one participant stated:

*“I used to drink but then, you know alcohol makes you forget.”*

And another reported:

*“I stopped taking medication when I started smoking cigarettes, weed, and drinking alcohol.”*

In addition to alcohol and substance use, some participants reported that mental health issues also interfered with their ability to adhere to treatment:

*“I just gave up on life, let me rather put it that way. I just forgot about life, I lost my marriage, I was sick, the man that I thought I had left me. When I heard that I am sick, I gave up. I just left everything.”*

Some participants also reported that they could not process the factual information given to them at counseling sessions because they were so upset about hearing their diagnosis. Thus, even though providers gave them objective reasons for their need to be adherent, they were unable to process this information. As one participant stated:

*Participant She said, that means I will be defaulting and that I will get sick and the virus will intensify because it intensifies when you stop taking treatment and I will get sick.*

*Interviewer Did she explain how the virus is contracted?*

*Participant I can’t remember, I don’t know, I don’t remember…I was still in shock, I couldn’t listen to anything.*

#### Relational influences

Participants described key moments in their relationships with others that impacted their adherence to cART. We have categorized these as “relational” influences on adherence since it was through these relationships that adherence was either supported or challenged. Key sub-themes in this relational realm included relationship issues around: (1) disclosure to others that they had HIV; (2) stigma; and (3) social support (both received and given).

Disclosure to others was an important part of relational influences on cART. Fear of disclosure or inadvertent disclosure of a person’s HIV status to others when medications were seen led to adherence challenges. The fear that knowledge of a participant’s HIV status would negatively affect relationships was ever present over the life course of the participants and a theme that was discussed throughout the interviews by all participants. They reported that they had to manage multiple disclosure events over the course of their lifetime, and that these disclosure events were times of great concern for them that often led to non-adherence. In particular, the fear over inadvertent disclosure—in which the tablets were seen by others who then presumed the person taking them had HIV—was described as a serious challenge to adherence.

As one participant reported:

*“It upsets me, you see when my friends come to visit, you know when its 8’oclock and its time for me to take the pills, sometimes I just stall and wait for them to leave because sometimes they can stay until 10’oclock and I don’t take the pills in front of them. I am scared to take them in front of them.”.*

And as another noted:

*“Whenever I get into a new relationship, I would never tell them that I am taking treatment.”* (Participant 6).

Stigma, and the anticipation of possible discrimination from others, was another relational reason for poor adherence to cART. Sometimes this stigma was internalized and led to a person either stopping their cART or not being willing to initiate cART at all. As one of the participants stated:

*“I never went on ARVs, because I said to myself, I never did anything wrong and how did I get this? I’d rather just die with it and then I never took any medication.”*

Although relationships with other people could be a source of stress and lead to non-adherence, nurutirng relationships were described by many participants as supporting adherence. This included relationships with health care providers, especially nurses. As one participant reported:

*“Then I realized, you know what? I can get better by them giving me this medication and in hospital the tablets were so big, I couldn’t swallow. So, three times the nurses caught the medication under my pillow. So, the nurse took me into the counsellor, there in the hospital. Then she said to me, ‘you know what, you’re so young. You’re beautiful. You can live your own life by doing things on your own, but I don’t want to see you again here, doing this’. I said no, you know what? I’ll think about this, and I’ll do what I have to do*.*”*

Family members were also reported to be key sources of adherence support. As one participant noted:

“*You know even my children remind me when its time to take the pills ‘Dad come drink your pills”. They would say, ‘it’s 8 o’clock’.”*

And another reported:

*“It was because my sister was encouraging me, and I would see her taking the pills. I would see that she would take them, and she was gaining weight and she was alright. So, I figured if I am to take this thing (pills) it would help me. So that’s when I started taking them.” (Participant 14).*

Relationships with other people living with HIV were also a source of social support that could facilitating adherence. One participant described how hearing from another woman living with HIV led to her (the participant’s) willingness to adhere to cART after prolonged periods of non-adherence:

*“She spoke and said, she had been on treatment for quite some time, she was telling her story and speaking about her life experiences…It was like God sent an angel to save me, I must stop trying to kill yourself and listen. This lady spoke about her experiences when I had not even asked her anything, … and said she is fine, and her family is supportive. The lady had answers to questions I had, and I needed those answers That was my day, and that woman made my day. I am alive because of her… She said, listen, live, life on earth is beautiful, live. You see, that’s what happened, that is what restored me.”*

Other important relationships that supported adherence included friends, people at work,and persons from church or religious groups.

In contrast to these relationships that supported cART adherence reported by study participants, others spoke of the loss of important social relationships and support as leading to their inability to adhere to treatment. One participant stated:

*“That’s when they found out that I have the virus and then my mother died. I didn’t have anyone to tell me how to take the pills.”*

Maintaining relationships with others—especially with healthcare providers—at times led to participants “covering up” their periods of non-adherence. They would report that they feared their relationships would be damaged if they let people know they were struggling to take their medications. Although it didn’t happen often, some participants even went so far as to pick up their prescriptions for their cART but not take them:

*“I used to collect them but not taking them because they used to annoy me with the phone calls asking me to come collect the pills…I don’t believe in the ARVs, personally, I don’t believe in them. So, there were times when I would collect the pills but not take them for about three months. But then I would be thinking that maybe they are right about the pills you see. I was on-and-off with them, because I had not decided what to believe about these pills.”*

#### Experiential influences

The third theme that emerged as significant in supporting or challenging adherence had to do with experiences the participants had while on treatment. We have categorized these as “experiential influences”. Participants described multiple important lived events in their treatment journey that impacted their ability to adhere to cART. Key sub-themes in the experiential realm had to do with the following: (1) experiencing or fearing negative physical feelings or symptoms, including fear of death (both at the time of diagnosis and during treatment with cART); (2) having adverse events; (3) experiencing discrimination or abuse; and (4) having competing health/social needs, including socioeconomic stressors. It also emerged during the data analysis that these experiences could be direct—that is they were actually faced by the participant him/herself—or indirect—that is they were witnessed or described as having happened to another individual.

Having been physically ill at the time of diagnosis and experiencing improvement when treatment was initiated and taken as prescribed was also reported to be a factor that promoted adherence. As one participant stated:

*“I had the abscess on my face… then I went to the clinic; when I got to the clinic, I requested an HIV test and they tested me and found out that I was HIV Positive. The abscesses, I had had them for a while and I would go to the clinic and they would give me medication and pills for the abscess, but they wouldn’t clear up and I would back again. Maybe this happened for a month, but when I started taking treatment, that’s when they cleared up.*

Watching or witnessing others die from HIV was a strong motivating factor to take cART. Many participants knew other individuals—either in their families or social groups—who were also living with HIV. Seeing the health of these individuals deteriorate due to not taking cART was reported to be something that encouraged individuals to continue taking cART:

*“When they told me, I said okay it’s like this and I had seen many of my friends die in front of me. We were a group of six friends, but I am now the only one who is left behind… they had already told me in hospital that if I don’t take my treatment I will die but if I take it and follow instructions, I will live as I am living.”*

After a period of not taking cART another participant stated:

*“I thought I was going to die, I basically felt like a dead man walking. I felt like I was nothing, I felt like I was nothing to people… I then figured but I am still alive, and I will be responsible for turning myself into nothing. I decided not to stop taking them (the ART) because I had concluded that maybe this is what they do to your body and if I stop taking them, that will mean that I am allowing this virus to spread in my body and kill me and the pills were meant to help. That’s why I did not stop taking them, because quitting the pills meant that the virus would kill me.”*

One of the reasons for poor adherence was the physical experience of adverse events. Almost all participants described a time when they felt that cART was making them sick. Although they tried to manage these side effects, they often became too difficult to bear and lead to a period of non-adherence. These adverse events were especially upsetting if they interfered with a person’s ability to be part of the activities and relationships that were important in their lives. As one participant reported:

*“If I take my pills and I get in an argument with someone, I will hear the voices and that affects me, that’s why I have decided to default… I am now a defaulter because of that.”.*

In contrast, some participants felt worse after they stopped taking their treatment. It was this experience of feeling ill off treatment that prompted them to start taking therapy again:

*“I had the pills as I did not take them for two weeks. I went to the clinic because of that…I could feel that my body was getting light, and sometimes I would feel like, you know when I take them, I do feel like something is happening in my body, you know if I eat and take them I do feel like something is happening in my body.”*

Many participants reported experiencing discrimination and abuse and these experiences were associated with poor adherence. As one participant stated:

*“My daughter’s mother-in-law and them used to call me such dirty words, “You are an HIV Bitch. You are a prostitute”. I used to just sit and cry because I never, I swear to God, I never ever slept with another man beside my husband… Then I said to myself, I’m not going to even take this medication; I’m just going to leave it. Then for three months I never came to the clinic, right, listen I never came to clinic.”*

Another set of experiences that made adherence challenging were those associated with competing health and social needs. Participants described socioeconomic difficulties, including hunger, loss of income (or fearing such loss), and the need to work as being a barriers to adherence to cART:

*“The problem that caused me to stop the treatment is lack of food at home… I cannot take a pill knowing that I will be hungry the whole night.”*

Another reported his loss of work as leading to the inability to adhere to cART: *“Do you think I was going to be able to perform my work duties? I lost my job because of the pills, I had to quit my job because I could see that I wasn’t coping. I would take them and try to force myself not to sleep at work because you can’t sleep on duty, you get fired for that.”*

Unanticipated travel for work was also reported as a reason for non-adherence. As one participant reported:

*“I got a part time plumbing job recently and I had to go to Swaziland for about 8 months. I had taken my pills with me because I get a 3 months’ supply, I thought we were going to stay in Swaziland for 2 months, but we were in there for 8 months and eventually my supply ran out and I had no idea where I was going to get my medication. This happened last year.” (Participant 17).*

#### Modifying and interacting lenses

Although the three thematic areas presented above accounted for most of the factors identified by participants as either supporting adherence or making it challenging, these areas were not discrete from one other and did not operate in completely separate spheres. Rather, they often interacted with and modified one another, leading to the balance favoring adherence or non-adherence. For example, a participant might have had the information that cART was the best way to keep HIV under control (factual realm), but felt worse after taking cART (experiential realm). The presence of a support person in his/her life who could guide the participant through the experience of adverse events might support adherence (relational realm), whereas a new relationship in which the partner was not aware of the participant’s HIV status might contribute to non-adherence (relational realm), especially if the participant skipped doses of cART in the past without any untoward consequences (experiential realm). Adherence could further be challenged if there are conflicts within this new relationship leading the person to seek comfort in alcohol or other substances (factual realm).

An example illustrating the interactions between the various experiences is in the quote below. The participant describes the indirect experience of seeing her sister die of HIV and how that experience shaped her willingness to take cART. In addition, she felt she could have supported her sister in taking cART, if only her sister had been able to disclose her HIV status.

*“I told myself that I can’t run away from the fact that this was now my status. I was ready and I had seen people dying and my sister also died because perhaps she didn’t have anyone to help her and had she received assistance she might have survived, maybe if she had told us we would have done things differently.”*

Another participant reported that his experience of aging would likely lead him to modify some of his relationships by disclosing his HIV status to people so they could help him in his adherence. He reported:

*“There isn’t anyone who has encouraged me, but I must tell them because I am getting older and I will start getting frail and I might not be as self-sufficient but if they know about my illnesses they will know when its time for me to take my pills and remind me.”*

## Discussion

Lifelong adherence to cART is necessary to support optimal health outcomes for people living with HIV. Although there have been some advances in supporting individuals adherence, much work needs to be done to address the multiple adherence challenges that individuals have to address on their treatment journey. Our study focused on exploring factors associated with periods of adherence and non-adherence within the same individual and revealed several important findings.

Perhaps the most important finding of the study was that it was a combination of facts, relationships with others, and both lived and witnessed experiences that contributed to participants’ ability to adhere to cART. The data analysis showed that participants reported most adherence counseling they received was focused on conveying facts to them, but when they described what supported or detracted from adherence, most of the time they talked about relationships and experiences. This suggests that there is a need to pivot from fact-based HIV counseling toward more interpretive and relational approaches. Although a variety of HIV counseling material has been developed and utilized in the treatment of HIV [10], a sizeable proportion focuses on conveying information or dispelling “myths and mystifications” about HIV. Some of this factual information bias likely stems from a tendency in Western biomedical approaches that prioritize the “rational” being, assuming that if presented with the right, seemingly “objective” information, human actors will make “correct” decisions that support their health care. However, there is also a hint of paternalism and colonialism in HIV counseling approaches that are fact-based, in that the provider is in a position of power and needs to educate the patient. Our data show the inherent misalignment of fact-based counseling with the factors reported by people to support or detract from adherence, which are much more based on their experiences (either direct or indirect) and relationships. In fact, experiences and relationships can often lead to a reassessment of facts that are conveyed in counseling sessions. HIV education and “treatment literacy” approaches that are primarily fact-based may, at their best, be considered a necessary part of cART counseling. On their own, however, they are rarely sufficient to support people living with HIV over the course of a lifetime.

Second, the data showed that relationships can have both a positive and a negative impact on adherence. It was interesting to see that, although there were measurable viral loads in each of the participants—signifying possible adherence challenges—most participants tried to present a narrative of consistent adherence to preserve important relationships. While it is possible that consistent adherence was common and detectable viral loads were due to malabsorption, viral “blips” that can be transient, or other infections that can increase HIV viral replication, it is likely that some participants who were struggling with adherence did not feel comfortable discussing this with their care providers because they wanted to maintain those relationships. This finding suggests there is a need to “normalize” adherence challenges as part of counseling and care sessions. Providers at all levels need to be non-judgmental about non-adherence and to recognize that this is a normal part of HIV treatment. Instead of repeatedly stressing the importance of adherence in cART, health care providers should encourage people to talk openly about adherence challenges to enable problem solving around periods of non-adherence. This is especially important in relation to adverse events, as discussion and support will facilitate adherence.

Another key relational influence on adherence that could be addressed by HIV programs is the important and ongoing process of disclosure. Any participants feared others finding out about their HIV and ART status through inadvertent disclosure. This led to participants both failing to collect their medication at the clinic, as well as not taking their medication. Part of this fear was the fear of disclosure and the uncertainty of how and what and when to disclose their HIV and ART status to friends and those with whom they were in an intimate relationship. Although disclosure is not addressed in the guidelines [11, 12], health care providers should be available and willing to discuss the challenges with disclosure and how it could be done at all interactions with patients.

Another important findings was that physical experiences played an important role in cART adherence. Many participants reported that the adverse events associated with cART were a barrier to adherence. Supportive relationships with people who could offer first-hand empathy and share real-world “tips” for overcoming the same challenges both individually or through “community” clubs has been reported to improve adherence [13, 14], a finding reported in our study as well. These relational factors may help mitigate some of the negative experiential influences on non-adherence and can also help re-orient people with adherence struggles to the fact that lifelong adherence is necessary even when adverse events happen. Training and compensating community members to formally provide such support is likely essential to facilitating cART adherence over the course of a lifetime. HIV programs should make the resources—both human and financial—available to ensure people who are struggling with adherence can access compassionate and well-informed peers as part of their routine care.

Many of those we interviewed delayed the initiation of cART until they became ill. Much has been published on the risk factors associated with delayed treatment initiation [15, 16] and the disadvantages of delayed treatment initiation[17]. Strategies to address treatment initiation delay have included same-day treatment initiation [18] and a revised counselling approach without mandatory multiple pre-initiation sessions, but to our knowledge, these have not been implemented at scale [19].

Other studies on problem-based models of adherence for cART support our findings [20]. In addition to counseling, some studies have found that economic support can improve adherence to cART [21]. Our findings that people reported significant adherence challenges when faced with economic needs and barriers also suggest more direct support to persons on cART over their treatment journey could improve optimal retention in care.

There are multiple limitations to this study. First, the study relied upon participant recall regarding periods of adherence and non-adherence, and there may have been recall bias that affected their narratives of these events. Second, there was a clear tendency on the part of participants to want to “please” the researchers by sharing stories of adherence, even when there were clear challenges to adherence. Although this tended to ease as the interviews progressed and trust was established, it may be that the participants under-reported the challenges they faced with adherence. Third, there was no testing for drug metabolites or objective measures of adherence other than viral loads that were assessed as part of routine treatment, and viral load measurements can be affected by variables other than adherence. Fourth, this was a small-scale qualitative study and further research will be needed to confirm the findings we report here. Finally, our theoretical model used to analyze the data was based on a review of the literature, but it may have missed important theoretical issues that were not explored—for example the role of gender in adherence to cART.

## Conclusion

In spite of these limitations, out study has several important findings that can be addressed through changes in practice. Persons on lifelong cART will experience challenges to adherence that can only be partially mitigated through fact-based counseling. Such counseling needs to be supplemented by strategies that focus on relationships and on physical experiences, both lived and witnessed. Non-adherence needs to be normalized within the health system and in interactions with providers at all levels so that persons experiencing adherences challenges do not cover them up to preserve their relationships and so that problem solving can occur. The factors that impact adherence may change over the course of a person’s treatment journey, and there need to be targeted approaches toward assessing and understanding adherence as a fluid variable. Finally, persons on cART may face many additional life challenges that can impact their ability to take their treatment as prescribed, including discrimination, abuse, hunger and loss of income. These factors also need to be addressed in an open and compassionate way so that people living with HIV can lead not only long but also healthy, happy and productive lives.

## Supplementary Information

**Additional file 1. Appendix 1**. In depth interview topic guide.

## Data Availability

The datasets used and/or analyzed during the current study are available from the corresponding author on reasonable request.
