# Supplementary material for: “I am alive because of her”: factors affecting adherence to combination antiretroviral therapy among people living with HIV in KwaZulu-Natal, South Africa
Source: BMC Infect Dis. 2022 Aug 8;22:680. doi: 10.1186/s12879-022-07667-x (PMC9361592; doi:10.1186/s12879-022-07667-x)
Supplement: Supplementary file 1 — Additional file 1. Appendix 1. In depth interview topic guide. [file 12879_2022_7667_MOESM1_ESM.docx]

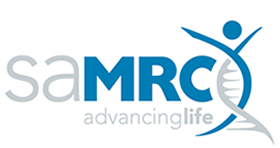


**APPENDIX 1: IN DEPTH INTERVIEW TOPIC GUIDE**

**The Treatment Journey of People Living with HIV**

**_____________________________________________________________________________________**

**Unique study no**: _______________________ **Gender: (M or F):** __________

**Interviewer:** ___________________________ **Date:** __­­­___________

**Questions:**

1. When were you diagnosed as having HIV?
2. Before you were diagnosed with HIV, can you remember what you knew about HIV?
3. Before you found out that you were living with HIV, do you remember what you thought about people who had HIV?

**Probes**

- Were you fearful of HIV?
- Did you avoid people with HIV? Why?
- Did people in the community gossip about so-and-so who they thought might have HIV?

1. Can you remember and describe how you were diagnosed with HIV?

**Probes**

- For women: Did you go to the clinic as you thought you were pregnant and that is when your HIV was diagnosed?
- For men and women: Did you start coughing and when you went to the clinic you were diagnosed with HIV and TB?
- Were you diagnosed with HIV the first time you went to the clinic? Or did you have to make numerous trips to the clinic with the same complaint before your HIV was diagnosed?
- Were you admitted to hospital at the time?
- Who told you the information?
- How did they tell you?

1. How did you feel when you were diagnosed with HIV?

**Probes**

- Was it disbelief/denial? (This can’t be. It can’t be me.)
- If so, how long did it take before you were able to accept this?
- Was it a shock?
- Do you wish it would have been done in a different way?
- Did you tell anyone? Why or why not?

1. How soon after your diagnosis did you start taking ART?

**Probes**

Straight away? Or was there a delay?

If there was a delay, why did you delay?

What does the ART do for you?

1. Do you remember the counselling sessions you had before you started ART?

**Probes**

- Were these sessions helpful?
- Did you learn much at these sessions about HIV and ART?
- Did you get information on how to manage your HIV?

1. What did you feel when you were advised to disclose your HIV status to someone else in the family/your partner?

**Probes**

- Was it disbelief/dismay? ‘I can’t do this’
- Was it not a big deal for you?
- Did anyone help you with this process of telling someone else?

1. Were you advised on how to disclose that you were HIV to your family/partner? If not, would you have liked support and advice on how to go about this?
2. Sometimes, when you start taking a course of medication you don’t feel yourself, but after a while, your body gets used to it. When you started taking ART, how did it make you feel?

**Probes**

- Did you stop taking it after you started as you felt so awful?
- How long did it take you to get used to it?
- Who did you talk to about the way you felt when you started taking ART? What did they do to help you?

1. And now, how do you feel about having to take ART every day?
2. You are here in hospital partly because you weren’t taking your ART. Why did you stop taking it?
3. You have been on ART for sometime, have there been previous occasions in which you stopped taking your treatment? Why?
4. What are the things or who are the people are support you as a person living with HIV who must take ART every day? How do these things support you?

**Probes**

- Does anyone from the family encourage you to go the clinic for your routine appointments?
- Is there a nurse who is particularly supportive of you?
- Does anyone tell you that you do not need to take your ART?

1. What are the things or who are the people that have made it difficult for you as a person living with HIV to take ART daily and attend the clinic for your routine visits? How have these things made it difficult for you?
2. Can you describe how the having HIV and taking ART has affected your life?

**Probes**

- Many people taking ART gain weight. How do you feel about gaining weight?
- Relationships: Have any of your relationships with your close family or friends been affected by having HIV? What happened?
- Stigma/discrimination? Has anyone or any group in your wider circle of acquaintances avoided you since you were diagnosed has HIV positive?
- Ability to work/provide for family?
